# Clinical Remarks upon Surgical Cases in the Buffalo General Hospital—Operation for Vesico-Vaginal Fistula—Entropion

**Published:** 1868-05

**Authors:** J. F. Miner


ART. II.—*Clinical Remarks upon Surgical Cases in the Buffalo General Hospital—Operation for Vesico-Vaginal Fistula—Entropion.* By J. F. Miner, M. D.

*Gentlemen:*—The effort to show you vesico-vaginal fistula and the operation for its cure, cannot be successful without great patience on your part, since what you can see is only to be observed through the speculum, and you will be obliged to organize in "Indian file," and pass singly in front, when the parts will be shown you. There is enough of interest in this case to insure your earnest attention, and reward you for all your patient, I may say, tedious effort.

*Vesico-Vaginal Fistula* is a communication between the bladder and vagina, and may be small and only seen with care, or, as in the present case, large and capable of admitting the finger readily into the bladder. Similar openings are liable to form communicating the bladder of the female with other organs—the uterus and rectum for instance—called vesico-uterine and vesico-rectal fistula. Vesico-vaginal fistula is much more frequent than any of the other forms, the common cause being the accidental laceration of the parts during parturition in consequence of the pressure of the child's head. The unskillful use of instruments have been known to cause it, but long continued pressure, as in very tedious labors, much more frequently results in sloughing and a communication such as you now observe. Some authors have stated that the vesico-uterine form of fistula is most frequent. I think, however, this must be a mistake.

*Diagnosis* of vesico-vaginal fistula is generally unattended with difficulty; careful inspection of the vagina will generally revea- the nature and extent of the lesion. By placing the patient so as to rest upon her knees and face, very little separating of the vaginal walls will admit of quite perfect inspection without the aid of instruments, and the point where urine escapes into the vagina will be plainly seen. The escape of urine through the vagina instead of through its natural channel will point out the nature of the lesion, no matter what may have been the causes operating to produce it. If there should be difficulty in finding the opening, on account of its small size, a catheter introduced into the urethra, by making the walls tense, may enable you to find it, when otherwise difficult.

*Prognosis* cannot be difficult when the true nature of the case is understood. If spontaneous recoveries have been known to occur when the accident was caused by a simple punctured wound, such termination in a case in any degree resembling the one you now observe, cannot be expected; it can only be remedied by operation.

This patient, Mrs. Odalum, is seventy-eight years of age, and she has suffered from this condition for thirty-eight years. Thirty-eight years ago, when this accident happened, caused by the birth of a child and instrumental delivery, it was scarcely regarded as possible to close these openings, the main and essential difficulty being the constant discharge of urine through the fistula, thus preventing adhesion of the edges. Improvements have been made in the plan of operation, and in the after treatment, so important as to remove most of the difficulties experienced by the older surgeons, and now we propose to attempt a restoration of parts after so long continuance, and in a very aged patient, an operation which was refused thirty years ago by a most capable surgeon, showing how great a change has taken place in the views of the profession, and the progress which has been made in perfecting the modes of operation.

There are but two points in the manner of operating to which I will call your special attention, viz.: paring the edges, and introduction of the sutures; do everything else in your own way, or after the advice of any one you choose to follow; but listen to one word from me upon those two points, vital to the success of your operation. Provided with suitable instruments, those now made for this purpose, consisting of a hook, three pairs scissors, short needles and silver wire, you may rely upon your own dexterity in their use, and proceed to do the operation as adroitly as you can. You can use such speculum as you find exposes the parts best, you can place your patient as you please, and change as you find convenient, but the two important steps in the procedure must be well taken or you fail of success. First then, you are to pare the edges of the aperture so deeply and thoroughly that the hardened cicatrix is removed, beveling off the edges towards the vaginal surface, making this paring more free, much more free, than you would be likely to do if unadvised upon this point. You will make this freshening with knife or scissors of whatever pattern you please, but above all, be sure to do it properly, safely, and thoroughly. There is no difficulty in doing it with the curved scissors now made for this purpose. The knife, in all forms yet proposed, is not as convenient. When this step has been completed, and the hemorrhage ceased, your sutures are to be introduced. Short needles, armed with silver wire, are to be inserted from before backwards. They are to extend down to the mucous membrane of the bladder and be placed at suitable distance back from the beveled edge. When all the sutures you need, have been introduced, placed not more than one-sixth or one-eighth of an inch apart, the wires can easily be drawn sufficiently tight to perfectly approximate the wound. They are not to be drawn too closely, lest you strangulate the parts, not to loosely lest you fail of complete and perfect coaptation and union. Now do not go off after any new or complicated method of tying up this wound; twist the wire gently until it is secure and sufficiently tight, and nothing better or more satisfactory can be desired. It is a simple, safe, and usually successful operation; it has been complicated by great refinement of description and parade of preparation, but paring the edges thoroughly and sewing them together nicely is all that is necessary to insure success. This patient is very old and infirm; success not so certain as in younger and more vigorous patients. Though but a few years more to live, still she thinks she is yet "worth mending," and as the operation involves but little risk, and, if successful, will add so much to her comfort and happiness, we have made it without hesitating as to its propriety; of its success or failure, I will inform you hereafter.*

*Entropion.*—We have an operation upon the eye-lid, which I suppose is the last of our operations before you during the present term. I will detain you but a few minutes longer, in describing its nature and showing you our method of cure. If the tarsal margins of the lids turn inwards against the surface of the eyeball, the rubbing of the eye-lashes against the conjunctiva causes constant irritation and discomfort. This turning in of the lid, from whatever cause, is called *entropion*, and in cases of any extent and severity can only be remedied by operation. You will observe that to remedy this case I have removed what I judge to be a sufficient amount of the skin of the lid and of the muscle beneath it, and after this unimportant hemorrhage has ceased, shall very accurately unite the wound with sutures. It is a matter of great importance to rightly estimate the amount of tissue to be removed; too much might cause the opposite deformity, turning outwards of the lid called *ectropion;* you will hence see the necessity of judging rightly in this, as in all other surgical operations.

After thanking you, gentlemen, for your constant and earnest attention to the clinical opportunities afforded you during the past term in the Buffalo General Hospital, and for all the repeated evidences you have given of appreciation of the value of such opportunities of observation, offered you mainly by the favor of the Trustees and Superintendent of this institution, allow me in conclusion to express the hope and expectation, that you will hereafter engage in the practice of surgery, with greater confidence and better preparation, and will remember these simple and plain clinical lessons in operative surgery.

---

At a recent trial in the United States Court in the city of Chicago, Judge Drummond sustained a physician in refusing to testify as an expert without having first received honorary fees therefor.—*Chicago Journal.*

---

* Examination of the patient May 14th, eight weeks after operation, shows the closure of the fistula complete. At no time after the sutures were adjusted has there been the least escape of urine. It now requires the most careful examination to detect the site of the old fistula, so perfect is the union. There is slight incontinence of urine, and it is necessary to urinate every two or three hours when up about the house, or there is apt to be involuntary escape of urine through the urethra; during the night there is no trouble, and she rests without disturbance.

